# Exposure to cooking fuels and birth weight in Lanzhou, China: a birth cohort study

**DOI:** 10.1186/s12889-015-2038-1

**Published:** 2015-07-28

**Authors:** Min Jiang, Jie Qiu, Min Zhou, Xiaochun He, Hongmei Cui, Catherine Lerro, Ling Lv, Xiaojuan Lin, Chong Zhang, Honghong Zhang, Ruifeng Xu, Daling Zhu, Yun Dang, Xudong Han, Hanru Zhang, Haiya Bai, Ya Chen, Zhongfeng Tang, Ru Lin, Tingting Yao, Jie Su, Xiaoying Xu, Xiaohui Liu, Wendi Wang, Yueyuan Wang, Bin Ma, Weitao Qiu, Cairong Zhu, Suping Wang, Huang Huang, Nan Zhao, Xiaosong Li, Qing Liu, Yawei Zhang

**Affiliations:** Department of Epidemiology and Biostatistics, West China School of Public Health, Sichuan University, 17 People’s South Road, Chengdu, Sichuan China; Gansu Provincial Maternity and Child Care Hospital, 143 North Road Qilihe District, 730050 Lanzhou, Gansu China; Department of Environmental Health Sciences, Yale School of Public Health, 60 College Street, New Haven, CT 06520 USA; Department of Epidemiology and Biostatistics, School of Public Health, Shanxi Medical University, 56 Xinjian South Road, Yingze District, Taiyuan, Shanxi China

**Keywords:** Cooking fuel, Birth weight, Low birth weight, Small for gestational age, China, Epidemiology

## Abstract

**Background:**

Early studies have suggested that biomass cooking fuels were associated with increased risk of low birth weight (LBW). However it is unclear if this reduced birth weight was due to prematurity or intrauterine growth restriction (IUGR).

**Methods:**

In order to understand the relationship between various cooking fuels and risk of LBW and small for gestational age (SGA), we analyzed data from a birth cohort study conducted in Lanzhou, China which included 9,895 singleton live births.

**Results:**

Compared to mothers using gas as cooking fuel, significant reductions in birth weight were observed for mothers using coal (weight difference = 73.31 g, 95 % CI: 26.86, 119.77) and biomass (weight difference = 87.84 g, 95 % CI: 10.76, 164.46). Using biomass as cooking fuel was associated with more than two-fold increased risk of LBW (OR = 2.51, 95 % CI: 1.26, 5.01), and the risk was mainly seen among preterm births (OR = 3.43, 95 % CI: 1.21, 9.74). No significant associations with LBW were observed among mothers using coal or electromagnetic stoves for cooking.

**Conclusions:**

These findings suggest that exposure to biomass during pregnancy is associated with risk of LBW, and the effect of biomass on LBW may be primarily due to prematurity rather than IUGR.

## Background

Low birth weight (LBW, <2,500 grams) is an important indirect cause of infant death worldwide [21] and more than half of neonatal deaths occur among LBW infants [2]. LBW has also been associated with delayed childhood development [30] as well as metabolic, infectious, and chronic diseases later in life [4, 28, 41]. It has been estimated that globally approximately 21 million infants are born with LBW each year. Incidence of LBW varies significantly across countries, ranging from 6 to 18 % with the majority of LBW infants occurring in Asia. Although China has a relatively low prevalence of LBW, it contributes significantly to the overall number of LBW worldwide given its large population size [39.]. Underlying biological contributors to LBW include prematurity (gestational age <37 weeks) and intrauterine growth restriction (IUGR) as measured by small for gestational age (SGA) [8].

Studies have linked LBW with certain environmental factors, such as maternal smoking and ambient air pollution [11, 19, 35, 38]. Exposure to household air pollution resulting from cooking fuels has also been suggested as an important cause of LBW in developing countries [26]. Several studies reported that exposure to biomass smoke was associated with an increased risk of LBW [1, 5, 15, 23, 33, 34, 36]. However, none of these studies have controlled for gestational age. It is unclear whether biomass smoke was associated with prematurity or intrauterine growth restriction. To advance the understanding of the relationship between cooking fuels and risk of LBW, we analyzed data from a birth cohort study conducted in Lanzhou, China to examine the association between specific cooking fuel types and the risk of LBW as well as SGA.

## Methods

The present study was based on data from a birth cohort study conducted during 2010-2012 at the Gansu Provincial Maternity & Child Care Hospital (GPMCCH), the largest maternity and childcare hospital in Lanzhou, China [27]. A total of 14,359 women aged 18 years or older, with no history of mental illness and gestational ages ≥20 completed weeks were eligible for the study. Of the eligible women, 3,712 refused to participate and 105 did not complete in-person interviews, which yielded 10,542 (73.4 %) women who participated in the study and completed in-person interviews. The distributions for maternal age and birth outcomes (i.e., multiple births, birth defects, and low birth weight) were similar for participants and non-participants. After excluding multiple births (*n* = 323), stillbirths (*n* = 40), birth defects (*n* = 253), births with missing information on birth weight (*n* = 30), and gestational age <22 weeks (*n* = 1), the analytic sample size was 9,895.

### All study procedures were ethically approved by the Yale University Human Investigation Committee and GPMCCH Institutional Review Board

Eligible women were informed of the study procedure upon their arrival at the hospital for delivery. After obtaining written consent, an in-person interview was conducted at the hospital either before or after delivery by trained study interviewers using a standardized and structured questionnaire. The questionnaire collected information regarding demographic factors, reproductive history, medical conditions and medication use, occupational exposures, and residential history.

Each participant was asked about current and past residences throughout her lifetime, including length of time residing at each residence (measured by dates of move-in and move-out). For each current and past residence, participants were asked about type of cooking fuel utilized (gas, coal, biomass, electromagnetic and others), ventilation practices while cooking, most frequently used types of cooking oil (e.g., canola oil, soybean oil, peanut oil, etc.), and the most commonly used cooking method (e.g., deep fried, pan fried, braised, etc.).

Information on birth outcomes and pregnancy complications were abstracted from medical records. Birth weight was measured in grams by trained professional nurses within the first hour of life. According to World Health Organization, LBW is defined as birth weight <2,500 grams, normal birth weight (NBW) is defined as birth weight between 2,500 grams and <4,000 grams, and macrosomia is defined as birth weight ≥4,000 grams [39].

Gestational age was calculated in completed pregnancy weeks based on the date of the last menstrual period. Information on last menstrual period was extracted from medical records. All self-reported last menstrual period dates were further verified by ultrasound examinations during antenatal care in the hospital. SGA, a measure of IUGR, is defined as a specified birth weight below the 10th percentile for gestational age of a reference standard; appropriate for gestational age (AGA) and large for gestational age (LGA) are specified as birth weights between the 10th and 90th and greater than 90th percentiles, respectively, for gestational age of the same reference standard [8]. We constructed SGA and AGA in our population based on the 2014 reference of Chinese infants from 28 to 44 gestation weeks [10]. Since there was no reference in the Chinese population for infants less than 28 weeks of gestation (*n* = 17), a United States national reference was applied for those with gestational ages between 22 and 27 weeks [12].

Chi-square or Fisher exact tests were employed to compare selected characteristics between LBW and NBW and between SGA and AGA. ANOVA tests were utilized to compare infants’ mean birth weights among women who utilized various types of cooking fuel during pregnancy. Unconditional logistic regression models were used to examine associations between cooking fuel and LBW and SGA. Potential confounding factors including maternal age, education, family income, parity, maternal weight gain during pregnancy, caesarean section, preeclampsia, vitamin supplements during pregnancy, smoking (active and passive), ventilation while cooking, and gestational age were included in the final models. Additional adjustment for maternal pre-pregnancy body mass index (BMI), alcohol consumption during pregnancy, gestational diabetes, infant gender, cooking temperature, and cooking oil type did not result in material changes in the observed associations and thus were not included in the final models. Odds ratios (OR) and 95 % confidence intervals (CI) were calculated using an unconditional logistic regression model. All analyses were performed using SAS 9.3 version (SAS Institute, Inc., Cary, NC).

## Results

In our cohort, 650 births were LBW and 8,581 were NBW while 746 births were SGA and 7,775 were AGA (Table 1). Compared to mothers with NBW or AGA infants, mothers of LBW or SGA infants were more likely to have lower education, lower family income, less weight gain during pregnancy, higher incidence of caesarean section and preeclampsia, exposure to passive/active smoking, poor cooking ventilation, and no vitamin supplementation during pregnancy. No differences in gestational diabetes, alcohol consumption during pregnancy, and infants’ gender were observed between LBW and NBW groups or between SGA and AGA groups. Women with LBW infants were more likely to be either younger (<25 years) or older (≥30 years) than women with NBW infants, while women with SGA infants were younger compared to women with AGA infants. Women with LBW or SGA infants were less likely to be primiparous compared to women with NBW or AGA infants. There was no difference in pre-pregnancy BMI between LBW and NBW groups; however, women with SGA infants were more likely to be classified as underweight (BMI ≤18.5 kg/m^2^) compared to women with AGA infants.

**Table 1:** Distribution of selected characteristics of the study population

Characteristic	LBW	NBW	*P*	SGA	AGA	*P*
	N (%)	N (%)		N (%)	N (%)	
Maternal age (years)						
<25	164(25.2)	1344(15.7)		161(21.6)	1256(16.2)	
25–29	227(34.9)	4205(49.0)		318(42.6)	3765(48.4)	
≥30	259(39.9)	3032(35.3)	<0.001	267(35.8)	2754(35.4)	<0.001
Highest education level						
High school or less	414(63.7)	3230(37.6)		399(53.5)	2972(38.2)	
College or above	219(33.7)	5196(60.6)	<0.001	333(44.6)	4656(59.9)	<0.001
Missing	17(2.6)	155(1.8)		14(1.9)	147(1.9)	
Family income (RMB per capita)						
<3000	436(67.1)	4233(49.3)		452(60.6)	3879(49.9)	
≥3,000	154(23.7)	3525(41.1)	<0.001	216(28.9)	3164(40.7)	<0.001
Missing	60(9.2)	823(9.6)		78(10.5)	732(9.4)	
Maternal pre-pregnancy BMI (kg/m^2^)						
≤18.5	135(20.8)	1822(21.2)		189(25.3)	1670(21.5)	
18.6–23.9	396(60.9)	5645(65.8)		445(59.7)	5122(65.9)	
≥24	79(12.1)	843(9.8)	0.078	77(10.3)	732(9.4)	0.010
Missing	40(6.2)	273(3.2)		35(4.7)	251(3.2)	
Maternal weight gain during pregnancy (kg)						
<15	346(53.2)	2541(29.6)		313(42.0)	2415(31.1)	
15–18.5	132(20.3)	2753(32.1)		215(28.8)	2468(31.7)	
>18.5	108(16.6)	2973(34.6)	<0.001	171(22.9)	2590(33.3)	<0.001
Missing	64(9.9)	314(3.7)		47(6.3)	302(3.9)	
Vitamin supplement during pregnancy						
No	150(23.1)	1234(14.4)		152(20.4)	1124(14.5)	
Yes	486(74.8)	7259(84.6)	<0.001	581(77.9)	6564(84.4)	<0.001
Missing	14(2.1)	90(1.0)		13(1.7)	87(1.1)	
Smoking (Active and passive)						
No	489(75.2)	6923(80.7)		560(75.1)	6272(80.7)	
Yes	161(24.8)	1658(19.3)	<0.001	186(24.9)	1502(19.3)	<0.001
Alcohol during pregnancy						
No	641(98.6)	8490(98.9)		735(98.5)	7693(98.9)	
Yes	2(0.3)	17(0.2)	0.882	2(0.3)	15(0.2)	0.989
Missing	7(1.1)	74(0.9)		9(1.2)	67(0.9)	
Gestational Diabetes						
No	647(99.5)	8513(99.2)		743(99.6)	7717(99.2)	
Yes	3(0.5)	68(0.8)	0.485	3(0.3)	58(0.8)	0.368
Preeclampsia						
No	539(82.9)	8388(97.8)		659(88.3)	7582(97.5)	
Yes	111(17.1)	193(2.2)	<0.001	87(11.7)	193(2.5)	<0.001
Parity						
Primiparous	379(58.5)	6375(74.3)		518(69.4)	5734(73.8)	
Multiparous	269(41.5)	2208(25.7)	<0.001	228(30.6)	2041(26.2)	0.012
Caesarean section						
No	298(45.8)	5473(63.8)		400(53.6)	4978(64.0)	
Yes	326(50.2)	3058(35.6)	<0.001	335(44.9)	2739(35.2)	<0.001
Miss	26(4.0)	50(0.6)		11(1.5)	58(0.8)	
Gender						
Boy	326(50.2)	4427(51.6)		369(49.5)	4067(52.3)	
Girl	322(49.5)	4138(48.2)		376(50.4)	3693(47.5)	
Missing	2(0.3)	16(0.2)	0.498	1(0.1)	15(0.2)	0.133
Ventilation						
No	271(41.7)	1646(19.2)		249(33.4)	1563(20.1)	
Yes	379(58.3)	6935(80.8)	<0.001	497(66.6)	6212(79.9)	<0.001

In our study population, 7,907 participants exclusively used gas as their cooking fuel, 358 exclusively used coal, 120 exclusively used biomas, and 487 exclusively used electromagnetic stoves (Table 2). A total of 1,023 participants used other fuels or multiple stoves (*n* = 168, 1.7 %) for cooking. A total of 140 participants used electromagnetic stoves and other stoves simultaneously, which accounted for approximately 22.3 % of women who ever used electromagnetic stoves. Women who used coal, biomass, or electromagnetic stoves were younger and less educated, and had lower family income and vitamin supplementation intake compared to women who used gas stoves. They were also more likely to be multiparous, have higher BMI, be diagnosed with preeclampsia, and report exposure to tobacco smoke during pregnancy. Women who used coal or biomass were more likely to have a higher caesarean section rate as compared to those who used gas. Infants delivered by women who used coal, biomass, or electromagnetic stoves were more likely to be LBW or SGA. Women who used coal, biomass, or electromagnetic stoves were also more likely to report poor cooking ventilation compared to those who used gas stoves.

**Table 2:** Distribution of selected characteristics of the study population by cooking fuel types

Characteristic	Gas	Coal	Biomass	Electromagnetic	Other	P*
	N (%)	N (%)	N (%)	N (%)	N (%)	
Birth weight						
LBW	371(4.7)	70(19.6)	42(35.0)	53(10.9)	114(11.1)	
NBW	6965(88.1)	270(75.4)	73(60.8)	408(83.8)	865(84.6)	<0.001
Macrosomia	571(7.2)	18(5.0)	5(4.2)	26 (5.3)	44(4.3)	
Birth weight						
SGA	509(6.4)	55(15.4)	22(18.4)	56(11.5)	114(10.2)	
AGA	6232(78.8)	264(73.7)	88(73.3)	377(77.4)	814(79.6)	<0.001
LGA	1166(14.8)	39(10.9)	10(8.3)	54(11.1)	105(10.2)	
Preterm						
No	7334(92.8)	267(74.6)	78(65.0)	417(85.6)	889(86.9)	
Yes	573(7.2)	91(25.4)	42(35.0)	70(14.4)	134(13.1)	<0.001
Maternal age (years)						
<25	1040(13.2)	136(38.0)	42(35.0)	226(27.9)	1580(22.1)	
25–29	3939(49.8)	108(30.2)	42(35.0)	438(43.1)	4737(42.8)	<0.001
≥30	2928(37.0)	114(31.8)	36(30.0)	359(29.0)	3578(35.1)	
Highest education level						
High school or less	2706(34.2)	302(84.4)	108(90.0)	277(56.9)	473(46.2)	
College or above	5111(64.7)	55(15.4)	10(8.3)	205(42.1)	466(45.6)	<0.001
Missing	90(1.1)	1(0.3)	2(1.7)	5(1.0)	84(8.2)	
Family income (RMB per capita)						
<3000	3788(47.9)	264(73.7)	99(82.5)	330(67.8)	495(48.4)	
≥3,000	3454(43.7)	58(16.2)	5(4.2)	137(28.1)	318(31.1)	<0.001
Missing	665(8.4)	36(10.1)	16(13.3)	20(4.1)	210(20.5)	
Maternal pre-pregnancy BMI (kg/m^2^)						
≤18.5	1640(20.7)	63(17.6)	16(13.2)	93(19.1)	208(20.3)	
18.6–23.9	5276(66.7)	228(63.7)	67(55.8)	336(69.0)	593(58.0)	
≥24	863(10.9)	36(10.0)	24(20.0)	49(10.1)	71(6.9)	<0.001
Missing	128(1.7)	31(8.7)	13(10.8)	9(1.8)	151(14.8)	
Maternal weight gain during pregnancy (kg)						
<15	2262(28.6)	158(44.1)	55(45.8)	204(41.9)	311(30.5)	
15–18.5	2573(32.6)	71(19.8)	20(16.7)	138(28.3)	248(24.2)	
>18.5	2910(36.9)	89(24.9)	25(20.8)	131(26.9)	298(29.1)	<0.001
Missing	162(2.9)	40(11.2)	20(16.7)	14(2.9)	166(16.2)	
Vitamin supplement during pregnancy						
No	963(12.2)	117(32.7)	49(40.8)	82(16.8)	255(24.9)	
Yes	6920(87.5)	239(66.8)	71(59.2)	403(82.8)	683 (66.8)	<0.001
Missing	24(0.3)	2(0.5)	0(0.0)	2(0.4)	85(8.3)	
Smoking (Active and passive)						
No	6416(81.1)	259(72.3)	91(75.8)	257(73.3)	844(82.5)	
Yes	1491(18.9)	99(27.7)	29(24.2)	130(26.7)	179(17.5)	<0.001
Alcohol during pregnancy						
No	7871(99.6)	357(99.7)	120(100.0)	487(100.0)	951(93.0)	
Yes	18(0.2)	0(0.0)	0(0.0)	0(0.0)	2(0.2)	0.697
Missing	18(0.2)	1(0.3)	0(0.0)	0(0.0)	70(6.8)	
Gestational Diabetes						
No	7828(99.0)	351(99.7)	118(98.3)	484(99.4)	1013(99.0)	
Yes	79(1.0)	1(0.3)	2(1.7)	3(0.6)	10(1.0)	0.539
Preeclampsia						
No	7710(97.5)	316(88.3)	101 (84.2)	464(95.3)	983(96.1)	
Yes	197(2.5)	42(11.7)	19(15.8)	23(4.7)	321(3.9)	<0.001
Parity						
Primiparous	5960(75.4)	186(52.0)	52(43.3)	339(69.6)	688(67.3)	
Multiparous	1947(24.6)	172(48.0)	68(56.7)	148(30.4)	335(32.7)	<0.001
Caesarean section						
No	4921(62.3)	194(54.2)	54(45.0)	307(63.0)	592(57.9)	
Yes	2927(37.0)	160(44.7)	65(54.2)	178(36.6)	417(40.8)	<0.001
Missing	59(0.7)	4(1.1)	1(0.8)	2(0.4)	14(1.3)	
Gender						
Boy	4162(52.6)	203(56.7)	69(56.7)	257 (57.5)	512(52.8)	
Girl	3732(47.2)	155(43.3)	51(43.3)	226(42.5)	509(46.4)	0.190
Missing	13(0.2)	0(0.0)	0(0.0)	4(0.0)	2(0.8)	
Ventilation						
No	751(9.5)	234(65.4)	98(81.7)	222(45.6)	713(69.7)	
Yes	7156(90.5)	124(34.6)	22(18.3)	265(54.4)	310(30.3)	<0.001

*The analysis did not account for missing data

Table 3 displays mean birth weight for each type of cooking fuel, as well as estimates for the difference in mean birth weight for biomass, electromagnetic, and coal stove users compared to gas stove users after controlling for potential confounders. The mean birth weight of the entire study population was 3,270 g (standard deviation, SD: 536 g). The mean birth weight of infants from households using biomass stoves was the lowest, followed by those using coal, electromagnetic, and gas stoves, respectively. Compared to households using gas, there were significant reductions in birth weight among households using coal (weight difference = 73 g, 95 % CI: 27, 120) or biomass (weight difference = 88 g, 95 % CI: 11, 164).

**Table 3:** Multiple linear regression model for mean birth weight of cooking fuel types

Fuel type	N	Mean ± SD(g)	Difference from gas^a^(g)	95 % CI
Gas	7907	3310.66 ± 499.16	0.00	
Coal	358	2970.40 ± 709.54	-73.31	-119.77 to -26.86
Biomass	120	2804.96 ± 803.89	-87.84	-164.46 to -10.76
Electromagnetic	487	3150.22 ± 613.10	-30.20	-69.02 to 8.63

^a^Adjusted for maternal age, education, and family income, and maternal weight gain, vitamin supplement during pregnancy, preeclampsia, caesarean section, parity, gestational week, smoking, and ventilation

The association between the various cooking fuels and LBW is presented in Table 4. Compared to using gas stoves for cooking, use of coal (OR = 1.92, 95 % CI: 1.37-2.69), biomass (OR = 3.74, 95 % CI: 2.35-5.94) or electromagnetic stoves (OR = 1.48, 95 % CI: 1.05-2.06) was associated with significantly increased risk of LBW. After additional adjustment for gestational age, only use of biomass for cooking remained statistically significant (OR = 2.51, 95 % CI: 1.26-5.01).

**Table 4:** Associations between type of fuel and risk of LBW

Fuel type	NBW	LBW	OR^a^(95 % CI)	OR^b^(95 % CI)
Gas	6965	371	1.00	1.00
Coal	270	70	1.92(1.37–2.69)	1.09(0.67–1.78)
Biomass	73	42	3.74(2.35–5.94)	2.51(1.26–5.01)
Electromagnetic	408	53	1.48(1.05–2.06)	1.14(0.71–1.83)

^a^Adjusted for maternal age, education, family income, maternal weight gain, vitamin supplement during pregnancy, preeclampsia, caesarean section, parity, smoking, and ventilation

^b^Additional adjustment for gestational week

In our study population, 910 (9.2 %) infants were preterm, with a mean gestational age of 33.8 (SD = 2.3) weeks. Among the preterm births, 776 were moderate to late preterm births (gestational age 32–36 weeks) and 134 were very preterm births (gestational age <32 weeks). We stratified the analysis by preterm and term births (Table 5). A significant association was observed only for those using biomass as cooking fuel among preterm births, but not term births (OR = 5.24, 95 % CI: 2.03–13.53 without adjustment for gestational age; OR = 3.43, 95 % CI: 1.21–9.74 with adjustment for gestational age). We further stratified our analyses by moderate to late preterm versus very preterm births. The observed associations were similar among moderate to late preterm births compared to total preterm births. No significant association was observed among very preterm birth, however, we may be underpowered to detect this association if it exists (*n* = 11 exposed cases).

**Table 5:** Associations between type of fuel and risk of LBW by preterm and term births

Fuel types	NBW	LBW	OR^a^(95 % CI)	OR^b^(95 % CI)
Term				
Gas	6668	102	1.00	1.00
Coal	239	10	1.00(0.48–2.09)	0.96(0.46–2.03)
Biomass	67	7	1.87(0.76–4.62)	1.85(0.72–4.71)
Electromagnetic	382	9	0.91(0.44–1.89)	0.84(0.40–1.76)
Preterm				
Gas	297	269	1.00	1.00
Coal	31	60	1.53(0.88–2.64)	1.26(0.67–2.37)
Biomass	6	35	5.24(2.03–13.53)	3.43(1.21–9.74)
Electromagnetic	26	44	1.47(0.84–2.58)	1.38(0.72–2.65)
Moderate preterm				
Gas	292	205	1.00	1.00
Coal	30	41	1.34(0.73–2.43)	1.25(0.64–2.41)
Biomass	6	24	4.32(1.61–11.58)	3.19(1.09–9.39)
Electromagnetic	25	35	1.58(0.87–2.87)	1.48(0.75–2.93)
Very preterm				
Gas	5	64	1.00	1.00
Coal	1	19	0.86(0.06–12.80)	0.85(0.06–12.69)
Biomass	0	11	—	—
Electromagnetic	1	9	0.41(0.03–5.52)	0.42(0.03–5.83)

^a^Adjusted for maternal age, education, family income, maternal weight gain, vitamin supplement during pregnancy, preeclampsia, caesarean section, parity, smoking, and ventilation

^b^Additional adjustment for gestational week

We further analyzed the association between cooking fuel types and the risk of SGA (Table 6). Compared to using a gas stove for cooking, use of biomass (OR = 1.22, 95 % CI: 0.70–2.08), coal (OR = 1.27, 95 % CI: 0.90–1.80), and electromagnetic stoves (OR = 1.27, 95 % CI: 0.93–1.74) were not significantly associated with the risk of SGA.

**Table 6:** Associations between type of fuel and risk of SGA

Fuel type	AGA	SGA	OR^a^(95 % CI)
Gas	6232	509	1.00
Coal	264	55	1.27(0.90–1.80)
Biomass	88	22	1.22(0.70–2.08)
Electromagnetic	377	56	1.27(0.93–1.74)

^a^Adjusted for maternal age, education, family income, maternal weight gain, vitamin supplement during pregnancy, preeclampsia, caesarean section, parity, smoking, and ventilation

## Discussion

This study is the first to investigate the associations between various types of cooking fuels and risk of LBW and SGA in the Chinese population. Our results support the hypothesis that use of biomass for cooking is associated with an increased risk of LBW compared to use of gas, and suggest that the association between biomass and LBW is likely due to prematurity but not IUGR.

Our study population primarily resided in an urban area. We noted a relatively high prevalence of gas stove use and low prevalence of biomass use compared to what we might expect in rural areas. The unbalanced prevalence of exposure may impact the accuracy of model estimation. However, the observed increased risk of LBW associated with biomass use was consistent with previous epidemiologic studies [1, 5, 15, 23, 33, 34, 36]. A cross-sectional study from India involving 14,850 infants found that using coal, kerosene, and biomass fuels for cooking was associated with a significant decrease in mean birth weight and increased risk of LBW [15]. Another Indian study including 47,139 infants reported that using biomass as cooking fuel was associated with a slightly increased risk of smaller size at birth; however, birth weight was measured indirectly, thus the results may be biased by the mother’s subjective recall [34]. A matched case-control study from the Gaza Strip including 446 births suggested that wood smoke was associated with an increased risk of LBW [1]. A retrospective population-based cohort study from Pakistan with a sample size of 634 suggested use of wood for cooking was associated with increased risk of LBW [33]. A Zimbabwean study based on 3,559 births reported that cooking with biomass was associated with reduced birth weight [23]. A study from Guatemala involving 1,717 women also showed a reduction in birth weight in association with using wood for cooking [5]. A birth cohort study of 9,604 participants reported that biomass was associated with increased risk of LBW and SGA [36]. A recent study from India that included 1,744 pregnant women found that wood fuel use was associated with non-significantly increased risk of LBW [40].

All of these published studies were conducted in areas where there was a very high prevalence of LBW, with rates as high as 33 %. In addition, the percentage of households that used biomass as a primary fuel source was also very high (up to 79 %), suggesting that the study populations had a very low socioeconomic status (SES). Malnutrition, which is common in these areas, plays an essential role in birth weight particularly in IUGR. However, neither these important confounders nor gestational age were controlled for in these prior studies.

Increased risk of LBW associated with biomass has been consistently reported in the literature, supporting our findings. Combustion of biomass fuel emits high concentrations of airborne particulate matter (PM) and toxic chemicals including carbon monoxide (CO), nitrogen dioxide (NO_2_), sulfur dioxide (SO_2_), and polycyclic aromatic hydrocarbons (PAHs) [17, 42]. When these pollutants are absorbed into the maternal bloodstream, the O_2_ content of maternal blood is reduced. Subsequently, O_2_ delivery to placenta is reduced, resulting in preterm delivery and subsequently LBW. Both epidemiological [9, 11, 13, 35, 38] and animal studies [25] have linked these pollutants to LBW. Therefore, it is biologically plausible that exposure to biomass smoke increases the risk of LBW.

Our study found that the effect of biomass on LBW was attenuated after adjusting for gestational age, which indicated a negative confounding effect. After stratification by preterm and term births, a significant association with biomass was only observed among preterm births but not term births. Our study also found no significant association between biomass and SGA, consistent with results from a recent study in India [40]. All these findings may suggest that prematurity rather than IUGR plays a major role in the association between biomass and LBW. An early study from southern India examined the effect of biomass use for cooking on LBW and SGA, and found that it was associated with both LBW and SGA, with a stronger association with LBW. In their study, the rates of LBW and SGA were approximately 33 % and 62 %, respectively [36], much higher than our study. These results suggested that if incidence of LBW was higher than 10 %, LBW was most likely caused by IUGR rather than prematurity, while if LBW incidence was less than 10 %, preterm infants constituted the majority of LBW [37]. The observed associations among preterm births need to be confirmed in future studies.

The literature has described a rapid rise in caesarean sections in China in the past decades. The current national rate is nearly 40 %, irrespective of geographic location or SES [16]. In our study population, the caesarean section rate was approximately 37.9 %, comparable to the national rate. Since the correlation between caesarean and preterm birth was relatively low (correlation coefficient = 0.08), caesarean section was unlikely a major contributor to preterm births.

A study from India reported a significant association between LBW and using coal for cooking, though they did not adjust for gestational age [15]. This was consistent with our study results prior to adjustment for gestational age; however, after controlling for gestational age the association diminished. A suggestive positive association between coal and SGA was also observed in our study. Because we cannot know if adjustment for gestational age would affect Epstein et al.’s results [15], it is unclear if the association with coal is unique to India or an artifact of uncontrolled confounding. It is possible that the level of pollutants released from combustion of coal in China is lower than those in India. A large number of studies have shown that concentrations of pollutants (i.e., CO and particular matters) released from coal are lower than those from biomass in China [14, 17, 22, 31, 32]. In addition, households in China usually use honeycomb briquettes with relatively high combustion efficiency, smoke is removed via chimney, and cooking generally occurs in a separate room or building [18]. As the majority of our study population came from an urban area, we would expect a higher percentage of households to be equipped with a chimney or hood in our study population. Our population is distinct from India, where most cooking stoves are simple (often made from mud as a U-shaped construction or three pieces of brick), have poor combustion efficiency, and are poorly ventilated [24].

We found that electromagnetic stoves were not associated with an increased risk of either LBW or SGA after controlling for gestational age. Electromagnetic stoves, more commonly known as induction cookers, use the electromagnetic induction principle to heat and cook food. Because the electromagnetic stove has many attractive features such as high energy efficiency, low noise, and no open flame, more families are beginning to replace coal, biomass, and gas stoves with induction stoves. To our knowledge, no previous study examined the association between electromagnetic stoves and LBW and SGA. Several studies have examined the association between electromagnetic fields (EMFs) and fetal growth. However, the results were inconsistent [29]. Occupational exposure to EMFs has been suggested to be associated with adverse birth outcomes (e.g., LBW, preterm, and birth defects) as reviewed by Robert [29]. Yet, studies have reported that among pregnant women, exposure to EMFs did not increase risk of LBW [3, 6, 20]. A non-significant but suggestive association between induction stove use during pregnancy and SGA in our study warrants further investigation.

The study population was recruited from the largest maternity and child care hospital in Lanzhou, the capital city of Gansu Province. The majority of study population came from Lanzhou City. Approximately 20 % of the remaining study population came from other cities and towns in Gansu Province. Although the study was hospital-based, which might impact generalizability, the LBW rate (6.2 %) in our study population was similar to the previously reported LBW rate (5.0 %) in all of Gansu Province [7].

Strengths and limitations should be considered when interpreting the study results. Information on birth weight was obtained from medical records and birth weight was measured in grams by trained professional nurses within the first hour of life, minimizing potential misclassification of the outcome. Information on gestational age was available, which allowed us to not only control for gestational age when studying the relationship with LBW, but also examine the association with SGA. One concern is that information on household heating source was not collected in the study. Since the majority of the study population came from urban areas where heating was centralized, the number of households using other fuels (such as coal or biomass) for heating was expected to be minimal. Generally households using coal or biomass for heating were likely to have lower SES; therefore we adjusted for SES (using education and family income as proxies) in our models. Lack of information regarding whether study participants were the primary person in charge of food preparation, number of meals cooked per day, and time spent in the kitchen might result in exposure misclassification. However exposure misclassification would likely be non-differential, if any, resulting in an underestimation of the observed associations. Given the various sources and factors that might influence indoor air pollution, it is important for future studies to employ more accurate methods, such as household or portable air quality monitors, to assess indoor air pollutants exposure.

## Conclusions

Our findings provided further support for the hypothesis that exposure to biomass is associated with an increased risk of LBW. Our study suggests that the effect of biomass smoke on LBW could be primarily due to prematurity rather than IUGR. Future studies are needed to clarify the biological mechanisms underlying the association. Additionally, a suggestive association between induction stove use and SGA warrants further investigation.

## Abbreviations

AGA: Appropriate for gestational age
CI: Confidence interval
CO: Carbon monoxide
EMF: Electromagnetic field
GPMCCH: Gansu Provincial Maternity & Child Care Hospital
IUGR: Intrauterine growth restriction
LBW: Low birth weight
LGA: Large for gestational age
NBW: Normal birth weight
NO_2_: Nitrogen dioxide
OR: Odds ratio
PAH: Polycyclic aromatic hydrocarbon
PM: Particulate matter
SD: Standard deviation
SES: Socioeconomic status
SGA: Small for gestational age
SO_2_: Sulfur dioxide
